# Upregulation of Spinal ASIC1 and NKCC1 Expression Contributes to Chronic Visceral Pain in Rats

**DOI:** 10.3389/fnmol.2020.611179

**Published:** 2021-01-11

**Authors:** Yong-Chang Li, Yuan-Qing Tian, Yan-Yan Wu, Yu-Cheng Xu, Ping-An Zhang, Jie Sha, Guang-Yin Xu

**Affiliations:** ^1^Jiangsu Key Laboratory of Neuropsychiatric Diseases and Institute of Neuroscience, Soochow University, Suzhou, China; ^2^Department of Gastroenterology, Jingjiang People’s Hospital, Jingjiang, China

**Keywords:** visceral pain, neonatal maternal deprivation, spinal dorsal horn, ASIC1, NKCC1

## Abstract

**Aims**: To determine whether acid-sensing ion channel 1 (ASIC1)–sodium-potassium-chloride cotransporter 1 (NKCC1) signaling pathway participates in chronic visceral pain of adult rats with neonatal maternal deprivation (NMD).

**Methods**: Chronic visceral pain was detected by colorectal distension (CRD). Western blotting and Immunofluorescence were performed to detect the expression and location of ASIC1 and NKCC1. Whole-cell patch-clamp recordings were performed to record spinal synaptic transmission.

**Results**: The excitatory synaptic transmission was enhanced and the inhibitory synaptic transmission was weakened in the spinal dorsal horn of NMD rats. ASIC1 and NKCC1 protein expression in the spinal dorsal horn was significantly up-regulated in NMD rats. Incubation of Amiloride reduced the amplitude of mEPSCs. Incubation of Bumetanide (BMT) increased the amplitude of mIPSCs. Intrathecal injection of ASIC1 or NKCC1 inhibitors reversed the threshold of CRD in NMD rats. Also, Amiloride treatment significantly reversed the expression of NKCC1 in the spinal dorsal horn of NMD rats.

**Conclusion**: Our data suggest that the ASIC1-NKCC1 signaling pathway is involved in chronic visceral pain in NMD rats.

## Introduction

Irritable bowel syndrome (IBS) is a functional gastrointestinal disease with a high population prevalence. It is characterized by chronic abdominal pain and altered bowel movements (Enck et al., 2016; Raskov et al., 2016). To date, there is a lack of effective treatment for the chronic visceral pain hypersensitivity associated with IBS due to the unclear underlying pathogenesis. Therefore, it is urgent to explore the detailed mechanisms leading to develop effective targets for the treatment of visceral pain. Recent studies have shown that spinal cord dysfunction may be one of the underlying pathogenesis of IBS (Zhao et al., 2017; Qi et al., 2019; Fan et al., 2020). As a primary center, the spinal cord plays an important role in the regulation of pain perception (Braz et al., 2014; Ji et al., 2019; Xu et al., 2019). However, the regulation of visceral pain sensitivity at the spinal cord level is rarely reported, so the mechanism remains largely unclear.

Acid-sensing ion channels (ASICs) are trimeric protein complexes composed of different subunit combinations, which are non-selective cationic channels and mainly expressed in the peripheral and central nervous systems (Lynagh et al., 2018; Yoder et al., 2018). ASIC1a, ASIC2a, and ASIC2b are mainly expressed in the central nervous system such as the brain and spinal cord, while ASIC1b and ASIC3 are mainly expressed in the peripheral nervous system (Lingueglia, 2007). To date, studies have shown that ASIC1 is involved in the regulation of various types of pain, such as inflammatory pain and neuropathic pain (Duan et al., 2007; Diochot et al., 2016; Li H.-S. et al., 2019; Wang et al., 2020). However, whether and ASIC1 is involved in the regulation of visceral pain at the spinal cord level remains unknown.

The cation-chloride co-transporters, Na^+^-K^+^-Cl^−^-Cl^−^-1 (NKCC1) and K^+^-Cl^−^-Cl^−^-2 (KCC2) play an important role in regulating Cl^−^ homeostasis in cells (Liu et al., 2019; Leterrier, 2020). The upregulation of NKCC1 is associated with the depolarized transformation of GABA reversed potential (E_GABA_) for some neurological diseases (Ben-Ari, 2017; Li C. et al., 2019). However, the role of NKCC1 in chronic visceral pain and how NKCC1 is regulated have not been fully understood.

In the present study, we used the well-established visceral hypersensitivity rat model, which is a suitable animal model to study pathophysiological characteristics of IBS-like (Du et al., 2019; Li et al., 2020), to verify the hypothesis that the upregulation of ASIC1 in the spinal dorsal horn contributes to the enhanced expression of NKCC1, thereby enhancing excitatory synaptic transmission of spinal dorsal horn neurons and resulting in chronic visceral pain in neonatal maternal deprivation (NMD) rats.

## Materials and Methods

### Animals

Adult male Sprague–Dawley (SD) rats (6–14 weeks old, 200–350 g) were housed at a constant temperature of 24 ± 2°C, 40–60% relative humidity, and a 12-h light-dark cycle in a clean-level animal facility at the Experimental Animal Center of Soochow University. The animals were provided by the Experimental Animal Center of Soochow University and approved by the Animal Care and Use Committee of Soochow University. Food and water can be obtained freely. Animals were used for behavioral experiments and to detect changes in electrophysiology and molecular expression. All experimental procedures were approved by the Animal Care Committee of the Soochow University and followed the guidelines of the International Association for the Study of Pain.

### Induction of Chronic Visceral Hyperalgesia

Chronic visceral pain was induced by NMD, as described previously (Du et al., 2019; Li et al., 2020). In short, from the 2 to 15 days of birth, the newborn SD male pups of the NMD group were placed in a separate box with an electric blanket for 3 h. After the separation period, the pups were placed back with their dams. Control pups and their dam were placed in the same cage without handling. On the 21st day after birth, the pups were weaned and separated from their dams. Experiments began at 6–7 weeks of age.

### Behavior Tests

Visceral hypersensitivity is determined by the colorectal distention (CRD) threshold as described previously (Du et al., 2019; Li et al., 2020). In brief, after the rats were lightly anesthetized with isoflurane, a self-made soft and flexible balloon (4 cm) was inserted into the rectum and colon 6 cm from the rat anus. The balloon was made by attaching the fingers of a surgical glove to an infusion hose. The infusion hose is firmly secured to the tail of the rat by tape. Rats were allowed to recover in a small and separate clear box for 30 min before CRD was performed. The balloon was slowly and uniformly inflated with a sphygmomanometer to know that the rat had a significant abdominal contraction reaction. At this time, the pumping was stopped to observe and the reading of the sphygmomanometer was read as the threshold of the CRD. Each rat was repeatedly measured three times at intervals of at least 3 min. All behavior tests were performed in a blinded manner.

### Slice Preparation

Both the control and the NMD groups (200–300 g) were deeply anesthetized with 4% chloral hydrate, and the rat abdomen was fixed upward and perfused. The spinal cord was quickly removed and fixed to a 3% agar block pre-prepared with a concave grain. The agar block with spinal cord tissue was placed into 31°C oxygenated (95% O_2_, 5% CO_2_) ice-cold Kreb solution with the following composition (in mM): 95 NaCl, 1.8 KCl, 1.2 KH_2_PO_4_, 0.5 CaCl_2_, 7 MgSO_4_, 26 NaHCO_3_, 15 Glucose, 50 sucrose, pH 7.2–7.4 (adjusted osmolarity with sucrose to 310–320 mOsm). The agar block was cyanoacrylate adhesive is fixed on a stage of the vibrating microtome. Several coronal sections (450 μm thickness) were cut with a Vibrating Microtome (Leica, VT1200S, Germany) while the spinal cord was placed in cold Krebs solution. Coronal sections were transferred to an oxygenated Krebs solution incubated at 31°C.

### Electrophysiological Recordings

After incubation for 1 h, one of the spinal cord slices was transferred to the recording grooves of nylon mesh, and the slice was fixed with a u-shaped nylon mesh. The slice at room temperature oxygenated recording liquid uniform continuous perfusion at 15 ml/min. Neurons used for recording in lamina II of spinal cord dorsal horn were visualized using infrared differential interference contrast (IR-DIC) video microscopy with a 40× magnification water-immersion objective (BX51WI, Olympus). Patch-clamp electrodes (5–10 MΩ tip resistance) were made using a P-97 puller (Sutter Instruments Company). Images of spinal cord slices reinforced with a CCD camera and can be clearly distinguished on a computer display. For recording action potentials (APs) and excitatory postsynaptic currents (EPSCs), the recording solution with the following composition (in mM): 127 NaCl, 2.4 CaCl_2_, 1.3 MgSO_4_, 1.2 KH_2_PO_4_, 1.8 KCl, 15 glucose and 26 NaHCO_3_ (pH = 7.2–7.3). The internal solution of the electrodes with the following composition (in mM): 133 K-gluconate, 0.6 EGTA, 8 NaCl, 2 Mg-ATP, and 0.3 Na-GTP, 10 HEPES (pH = 7.2–7.3). Spinal dorsal horn neurons in lamina II using a whole-cell patch-clamp recording. The tip of the patch electrodes was slowly lowered to the surface of the slice by a micromanipulator (MP-225). After Giga ohm seals (usually 2–6 GΩ) were formed and the whole-cell configuration was obtained, neurons were tested if the resting membrane potential was more negative than −50 mV and direct depolarizing current injections (40–160 pA, step 40 pA, duration 500 ms) evoked APs overshooting 0 mV when recording EPSC and Aps (Yang and Li, 2000). Miniature EPSCs (mEPSCs) were recorded by adding TTX (1 μM) to the recording solution. For recording inhibitory postsynaptic currents (IPSCs), the recording solution with the following composition (in mM): 124 NaCl, 24 NaHCO_3_, 1.2 NaH_2_PO_4_, 2.5 KCl, 2 MgSO_4,_ and 2 CaCl_2_, 5 HEPES, 12.5 glucose. For recording sIPSCs the internal solution with the following composition (in mM): 140 CsCl, 2 Na-ATP, 1 EGTA, 2 MgCl_2_, 0.3 Na-GTP, 2 Na-ATP, 0.3 Na-GTP, 5 QX314, 10 HEPES, pH was adjusted to 7.2 with CsOH. Excitatory synaptic transmission was blocked by D-AP5 (30 μM) and CNQX (10 μM) when recording sIPSCs. Miniature IPSCs (mIPSCs) were recorded by adding TTX (1 μM) to the recording solution. For recording EPSCs and IPSCs, the holding potentials were −70 mV. Amiloride (100 μM) and Bumetanide (BMT; 20 μM) were used to block ASIC1 receptors and NKCC1 receptors, respectively. All drugs were dissolved in ACSF on the day of the experiment and added by perfusion. All data collection by Digidata 1440A interface, MultiClamp 700B amplifier, and pClamp10 software (Molecular Devices, Axon, USA). Data were filtered and sampled at 5 kHz with a Bessel filter amplifier. Data were stored on a computer for offline analysis. In all electrophysiological data, n represents the number of neurons recorded.

### Western Blotting

After deep anesthesia, the spinal dorsal horn tissue was quickly dissected in an ice-cold and oxygenated ACSF, containing (in mM): 95 NaCl, 1.8 KCl, 1.2 KH_2_PO_4_, 0.5 CaCl_2_, 7 MgSO_4_, 26 NaHCO_3_, 15 Glucose, 50 sucrose, pH 7.2–7.3 (adjusted osmolarity with sucrose to 310–320 mOsm). Spinal dorsal horn tissues from control and NMD rats were disrupted to homogenate by a sonicator. The homogenate was placed on ice for 2 h and centrifuged at 15,000 rpm for 30 min at 4°C, and the supernatant suspension was retained for protein detection. The amount of protein was detected by the BCA protein assay kit (MultiSciences, Hangzhou, China). The protein was denatured in a 75°C constant temperature water bath for 10 min, and the protein loading was loaded into 10% sodium dodecyl sulfate-polyacrylamide gels (Bio-Rad, Hercules, CA, USA) and transported to polyvinylidene difluoride membranes (Millipore). After the transfer, the membranes were blocked with a Tris-HCL buffer solution (TBS, 50 mmol/l Tris-HCl, 133 mmol/l NaCl, pH = 7.4) containing 5% skim milk at room temperature for 2 h, and incubated with specific antibodies for 12–24 h at 4°C. Antibodies included anti-GAPDH (1:2,000, Goodhere Biotechnology, Hangzhou, China), anti-ASIC1 (1:1,000, Alomone Labs, Jerusalem, Israel), anti-ASIC2 (1:1,000, Alomone Labs, Jerusalem, Israel), anti-NKCC1 (1:500, Abcam, UK) and anti-KCC2 (1:1,000, Abcam, UK). After washed in TBS containing 0.5% Tween-20 (TBST), the membranes were incubated with horseradish peroxidase-conjugated anti-mouse (1:2,000, Multi Sciences, Hangzhou, China) or anti-rabbit IgG secondary antibodies (1:2,000, Jackson ImmunoResearch Laboratories, PA, USA) in TBS containing 1% milk at room temperature. Finally, protein bands were quantified by enhanced chemiluminescence (Tanon, Shanghai, China), and optical density analysis was performed. All images were taken with ImageJ software (Bio-Rad, CA, USA).

### Immunofluorescence

Immunofluorescent labeling was performed as previously described (Sun et al., 2019). Rats were transcardially perfused with 0.9% normal saline and 4% paraformaldehyde, and the spinal cord was removed and post-fixed for 2 h. The thickness of the spinal cord slice 20 μm was processed. After blocking in phosphate-buffered saline (PBS) containing 7% normal donkey serum, 0.3% Triton X-100, and 0.05% sodium azide at room temperature for 1 h, the slices were incubated with primary antibodies, including anti-ASIC1 (1:50, Alomone Labs, Jerusalem, Israel), anti-NKCC1 (5 μg/ml, Developmental Studies Hybridoma Bank, Iowa City, IA, USA), anti-NeuN (1:50, Merck Millipore, Darmstadt, Germany), anti-GFAP (1:100, Cell Signaling Technology, Danvers, MA, USA) or anti-CD11b (1:50, Bio-Rad, CA, USA) overnight at 4°C. After wash, the slices were then incubated in secondary antibody included Alexa Fluor 488 (1:500, Molecular Probes New York) or Alexa Fluor 555 (1:100, Molecular Probes New York) for 2 h at room temperature.

### Quantitative Real-Time PCR

Total RNA was extracted from the dorsal horn of the spinal cord in control and NMD rats using Trizol Reagent (Ambion, TX, USA). cDNA was synthesized using a reverse transcription kit (Transgen Biotech, Beijing, China) following the manufactories instruction. The primer sequences used in qPCR are shown in Table 1. Negative control reactions were performed by omitting the cDNA temple. The relative expression level for each target gene was normalized *via* the 2^−ΔΔCt^ method.

**Table 1:** The primer sequences used in qPCR.

Primers	Sequences (5′ to 3′)
GABA_A_α1-F	GTCCATGATGGCTCAAACCG
GABA_A_α1-R	GGGCTGTCCATAGCTTCTTCC
GABA_A_α2-F	TTGCTGTACACCATGAGGCTT
GABA_A_α2-R	CTTCCGAGGTCGTGTAAGCA
GABA_A_α3-F	GGTTAGAAGGCACGCCCATA
GABA_A_α3-R	TGGGAGAGAGGCCTCCAATA
GABA_A_α4-F	TGGATTTGGGGGTCCTGTTAC
GABA_A_α4-R	ACCTCAGGATTTCAATGGGGC
GABA_A_α5-F	GCTCCAGTGCCATCCCTTAT
GABA_A_α5-R	GGCATTTGTGAAAAGCCAAAGTG
GABA_A_α6-F	GACAACTTGCTGGAGGGCTA
GABA_A_α6-R	TCCATTGTGTACTCCATCTCCA
GABA_A_β1-F	CGAGAGAGTTTGGGGCTTCTC
GABA_A_β1-R	GCTGGGTTCATTGGAGCTGTG
GABA_A_β2-F	ATGTCAACAAGATGGACCCACA
GABA_A_β2-R	ATGCTGGAGGCATCATAGGC
GABA_A_β3-F	CTGTACGGGCTCAGGATCAC
GABA_A_β3-R	ACCTGTGGCGAAGACAACAT
GABA_B1_-F	ACGTGGCTTGGCATTTTCTATG
GABA_B1_-R	TCATGGTCACAGGAGCAGTG
GABA_B2_-F	ACTACACAGACCACACGCTG
GABA_B2_-R	TCGGACCCCTGGAACCTTAT
NKCC1-F	GCAAGACTTCAACTCAGCCA
NKCC1-R	TCCATCATCAAAAAGCCACCAG
KCC2-F	CCATCTACGCAGGGGTCATC
KCC2-R	GGCGGGAGGAACAGAATAGG
GAPDH-F	GGTTGTCTCCTGCGACTTCA
GAPDH-R	TGGTCCAGGGTTTCTTACTCC

### Drug Administration

For behavioral experiments, amiloride, psalmotoxin 1 (PcTX1), and BMT or dimethyl sulfoxide (DMSO) was intrathecally injected into the L4–L5 spinal cord level of rats *via* a microsyringe. The rats were given persistent anesthesia with isoflurane and placed on a board with the abdomen facing down and the spine L3–L5 segments curved (De la Calle and Paíno, 2002; Du et al., 2019). The tail swing of the rats during intrathecal injection was considered successful. The drug was slowly injected and the needle was left for at least 30 s to ensure that the drug did not reflux. The concentration of the drug used in this study is based on our previous studies and our previous work with Lab (Wang et al., 2020).

### Data Analyses

All data are presented as mean ± SEM. Error bars in the figures stand for SEM. Statistical testing was performed using Origin 8 (Origin Lab, Inc., Northampton, MA, USA). Normality was first checked for all data before analysis. Significance was determined using the Mann–Whitney test, two-sample *t*-test, Mann–Whitney test following Friedman ANOVA, or Tukey’s *post hoc* test following two-way repeated-measures ANOVA. Statistically significant was considered when* P* < 0.05.

## Results

### NMD Increased Excitability of Spinal Dorsal Horn Neurons in Visceral Pain Condition

**Figure 1:**
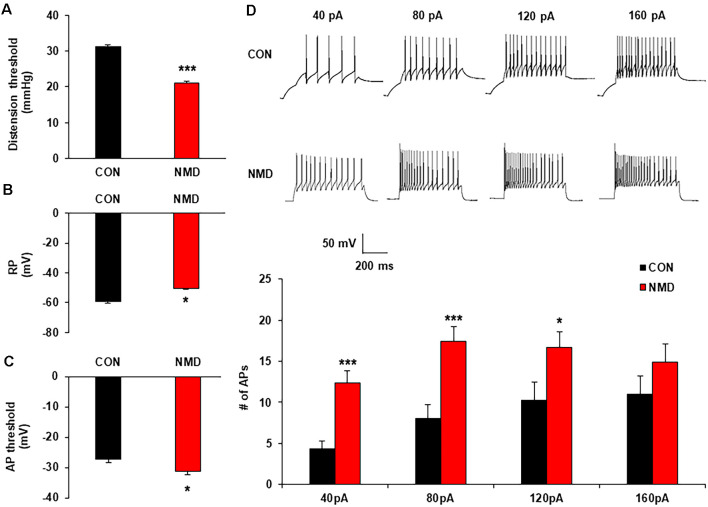
Neonatal maternal deprivation (NMD) reduced visceral pain threshold and enhanced the neuronal excitability. **(A)** NMD rats developed chronic visceral pain (***P < 0.001, *n* = 6 rats for each group, Tukey *post hoc* test following two-way repeated measures ANOVA). **(B)** NMD depolarized the resting membrane potential (RP; *P < 0.05, *n* = 17 cells for CON and *n* = 15 cells for NMD, two sample *t*-test). **(C)** NMD hyperpolarized the threshold of action potential (AP) in NMD rats when compared with CON (*P < 0.05, *n* = 10 cells for CON and *n* = 17 cells for NMD, two sample *t*-test). **(D)** NMD significantly enhanced the frequency of AP method under 40, 80, and 120 pA ramp current stimulation (*P < 0.05, ***P < 0.001, *n* = 17 cells for CON and *n* = 15 cells for NMD, two sample *t*-test).

The measurement of chronic visceral pain in the 6-week control and NMD rats was indicated by the threshold of abdominal withdrawal reflex in response to colorectal distention (CRD) as previously demonstrated (Wu et al., 2019). The CRD threshold of NMD rats was significantly reduced when compared with CON (Figure 1A, ***P < 0.001, Tukey *post hoc* test following two-way repeated-measures ANOVA). The data confirms the idea again that NMD might develop chronic visceral hyperalgesia in adult rats (Xiao et al., 2016; Du et al., 2019; Li et al., 2020).

To determine the effects of NMD on the excitability of spinal dorsal horn neurons in the T13-L2, whole-cell recording by patch-clamp was performed. The resting membrane potential of neurons in NMD rats was significantly increased (Figure 1B; *P < 0.05, two-sample *t*-test), but the threshold of AP emission was reduced compared with CON rats (Figure 1C, *P < 0.05, two-sample *t*-test). Additionally, the number of APs in NMD T13-L2 spinal dorsal horn neurons was significantly increased under 40, 80, and 120 pA current stimulation as compared with CON rats (Figure 1D, *P < 0.05, ***P < 0.001, two-sample *t-test*). These data indicate that the excitability of spinal dorsal horn neurons of T13-L2 in NMD rats with visceral hypersensitivity.

### NMD Enhanced sEPSCs but Decreased sIPSCs of Spinal Dorsal Neuron

**Figure 2:**
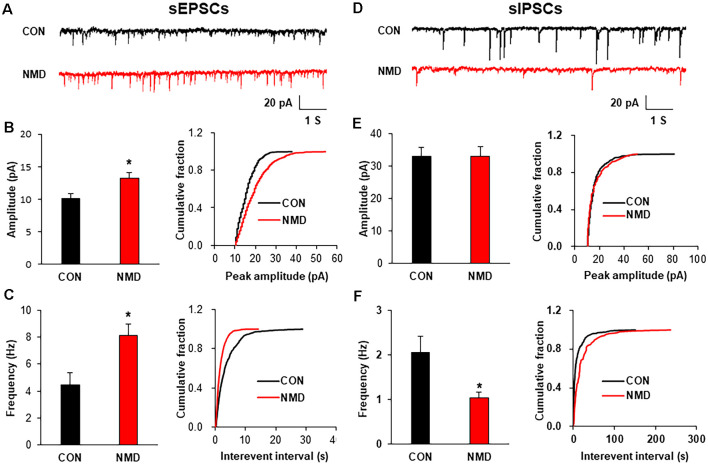
NMD enhanced spinal sEPSCs and reduced sIPSCs. **(A)** Representative traces of sEPSCs recorded in the spinal dorsal horn neurons of CON and NMD rats at T13-L2. **(B)** Bar plots and cumulative probability distributions of the amplitude of sEPSCs recorded in the spinal dorsal horn neurons of CON and NMD rats at T13-L2 (*P < 0.05, *n* = 9 cells for CON and *n* = 18 cells for NMD, two-sample *t*-test). **(C)** Bar plots and cumulative probability distributions of the frequency of sEPSCs recorded in the spinal dorsal horn neurons of CON and NMD rats at T13-L2 (*P < 0.05, *n* = 9 cells for CON and *n* = 18 cells for NMD, two-sample *t*-test). **(D)** Representative traces of sIPSCs recorded in the spinal dorsal horn neurons of CON and NMD rats at T13-L2. **(E)** Bar plots and cumulative probability distributions of the amplitude of sIPSCs recorded in the spinal dorsal horn neurons of CON and NMD rats at T13-L2 (*P* > 0.05, *n* = 7 cells for CON and *n* = 8 cells for NMD, two-sample *t*-test). **(F)** Bar plots and cumulative probability distributions of the frequency of sIPSCs recorded in the spinal dorsal horn neurons of CON and NMD rats at T13-L2 (*P < 0.05, *n* = 7 cells for CON and *n* = 8 cells for NMD, two-sample *t*-test).

To explore the mechanism of increased neuron excitability, patch-clamp recording in spinal slices was performed to determine the synaptic transmission. The representative traces of sEPSCs showed that excitatory synaptic transmission was enhanced in spinal dorsal neurons of NMD rats compared with CON rats (Figure 2A). Both amplitude and frequency of sEPSCs in NMD rats were significantly increased compared with CON rats (Figures 2B,C, *P < 0.05, two-sample *t-test*). Additionally, the representative traces of sIPSCs showed a decreased inhibitory synaptic transmission in NMD rats compared with CON rats (Figure 2D). Notably, the frequency of sIPSCs in NMD rats was significantly decreased but the amplitude was not changed (Figures 2E,F, *P < 0.05, two-sample *t*-test). These results suggest that the enhanced sEPSC and decreased sIPSC might contribute to the increased neuron excitability of NMD rats.

### ASIC1 Expression Was Up-Regulated in the Spinal Superficial Neurons

**Figure 3:**
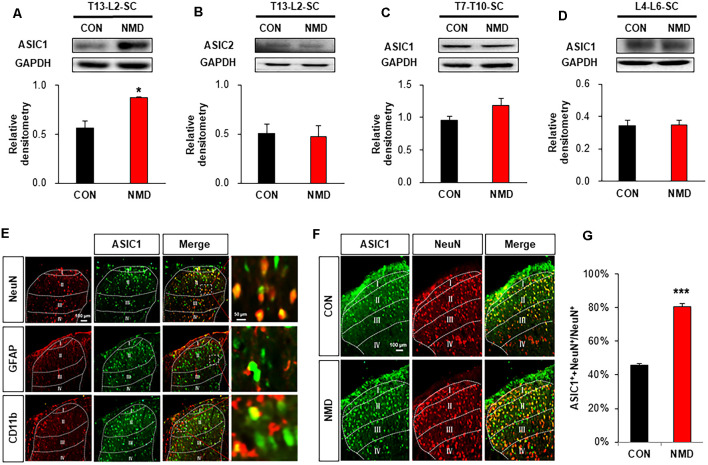
NMD enhanced acid-sensing ion channel 1 (ASIC1) expression. **(A)** Expression of ASIC1 in the spinal dorsal horn of T13-L2 in NMD rats compared with CON rats (*P < 0.05, *n* = 5 rats for CON and *n* = 4 rats for NMD, two-sample *t*-test). **(B)** Expression of ASIC2 in the spinal dorsal horn of T13-L2 in NMD rats compared with CON rats (*P* > 0.05, *n* = 5 rats for CON and *n* = 4 rats for NMD, two-sample *t*-test). **(C)** Expression of ASIC1 in the spinal dorsal horn of T7–T10 in NMD rats compared with CON rats (*P* > 0.05, *n* = 4 rats for each group, two-sample *t*-test). **(D)** Expression of ASIC1 in the spinal dorsal horn of L4–L6 in NMD rats compared with CON rats (*P* > 0.05, *n* = 4 rats for each group, two-sample *t*-test). **(E)** Location of ASIC1 in the spinal dorsal horn of T13-L2 in NMD rats. **(F)** Co-localization of ASIC1 and NeuN in the spinal dorsal horn of T13-L2 in CON and NMD rats. **(G)** Expression of ASIC1 in the spinal dorsal horn of T13-L2 in CON and NMD rats (***P < 0.001, *n* = 3 rats for each group, two-sample *t*-test).

The expression of ASICs was detected by western blotting to investigate the mechanism of enhanced excitatory synaptic transmission. As shown in Figure 3A, the protein levels of ASIC1 was significantly increased in the T13-L2 spinal dorsal horn of NMD rats compared with CON rats (Figure 3A, *P < 0.05, two-sample *t*-test), but the protein expression of ASIC2 was not altered (Figure 3B, *P* > 0.05, two-sample *t*-test). We also determined the protein levels of ASIC1 in the non-colonic segment. The ASIC1 expression was not changed in T7–T10 and L4–L6 spinal dorsal horn of NMD rats compared with CON rats (Figures 3C,D, *P* > 0.05, two-sample *t*-test). Further, an immunofluorescence assay was performed to investigate the expressed location of ASIC1. The results of immunofluorescence showed that ASIC1 was mainly expressed in neurons rather than glial cells of the spinal dorsal horn (Figure 3E). Immunofluorescence was performed to further evaluate the expression of ASIC1 in CON and NMD rats (Figure 3F). The results showed that the expression of ASIC1 in NMD rats increased significantly compared with CON rats (Figure 3G; ***P < 0.001, two-sample *t-test*).

### Amiloride Relieved Chronic Visceral Pain of NMD Rats *via* a Postsynaptic Mechanism

**Figure 4:**
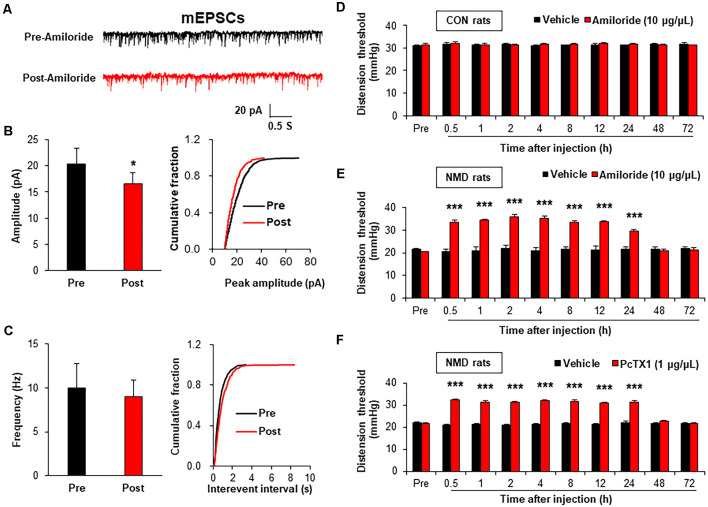
Amiloride reduced spinal mEPSCs and enhanced visceral pain threshold in NMD rats. **(A)** Representative traces of mEPSCs were recorded in spinal dorsal horn neurons of NMD rats before and after incubation of Amiloride. **(B)** Bar plots and cumulative probability distributions of the amplitude of mEPSCs recorded in spinal dorsal horn neurons of NMD rats before and after incubation of Amiloride (*P < 0.05, *n* = 5 cells for each group, two-sample *t*-test). **(C)** Bar plots and cumulative probability distributions of the frequency of mEPSCs recorded in spinal dorsal horn neurons of NMD rats before and after incubation of Amiloride (*P* > 0.05, *n* = 5 cells for each group, two-sample *t*-test). **(D)** Behavioral changes of CON rats after intrathecal injection of Amiloride (10 μg/μl) for seven consecutive days (*P* > 0.05, *n* = 6 rats for each group, Tukey *post hoc* test following two-way repeated-measures ANOVA). **(E)** Behavioral changes of NMD rats after intrathecal injection of Amiloride (10 μg/μl) for seven consecutive days (***P < 0.001, *n* = 6 rats for each group, Tukey *post hoc* test following two-way repeated-measures ANOVA). **(F)** Behavioral changes of NMD rats after intrathecal injection of PcTX1 (1 μg/μl) for seven consecutive days (***P < 0.001, *n* = 6 rats for NMD + DMSO group and *n* = 8 rats for NMD + PcTX1 group, Tukey *post hoc* test following two-way repeated-measures ANOVA).

It is well known that the change in frequency of mEPSCs reflects the presynaptic changes at the transmitter release site, whereas the change in amplitude of mEPSCs reflects the changes at the postsynaptic membrane (Wyllie et al., 1994; Fu and Neugebauer, 2008). Therefore, the mEPSCs were measured before and after incubation with Amiloride (100 μM; Wang et al., 2020), a known antagonist of ASIC1, to assess the effect of ASIC1 on presynaptic or postsynaptic sites. The representative trace of a typical neuron showed that suppression of ASIC1 by Amiloride decreased the amplitude of mEPSCs recorded in spinal dorsal horn neurons of NMD rats (Figure 4A). The Amplitude of mEPSCs in Post-Amiloride was significantly decreased compared with Pre-Amiloride (Figure 4B, *P < 0.05, two-sample *t*-test), but the frequency of mEPSCs in Post-Amiloride was not altered (Figure 4C, *P* > 0.05, two-sample *t*-test). To further determined the role of ASIC1 in chronic visceral pain of NMD rats, a known ASIC1 antagonist Amiloride was intrathecal injected daily for a consecutive week (Wang et al., 2020). The threshold of colorectal distention in CON + Amiloride groups was not altered compared with CON + DMSO groups (Figure 4D, *P* > 0.05, Tukey *post hoc* test following two-way repeated-measures ANOVA). The threshold of colorectal distention in NMD + Amiloride groups was markedly increased compared with NMD + DMSO groups (Figure 4E, ***P < 0.001, Tukey *post hoc* test following two-way repeated-measures ANOVA). To further detect which ASIC1 subunit regulates chronic visceral pain in NMD rats, we performed behavioral tests on NMD rats after intrathecal injection of PcTX1 (1 μg/μl; Aissouni et al., 2017) for a week. The threshold of colorectal distention in NMD + PcTX1 groups was significantly increased compared with NMD + DMSO groups (Figure 4F, ***P < 0.001, Tukey *post hoc* test following two-way repeated measures ANOVA). The results of NMD rats injected PcTX1 were consistent with that of Amiloride. These results indicate that ASIC1 enhanced the excitatory spinal synaptic transmission *via* a postsynaptic mechanism eventually contributing to chronic visceral pain.

### NMD Increased NKCC1 Expression in the Spinal Dorsal Horn

**Figure 5:**
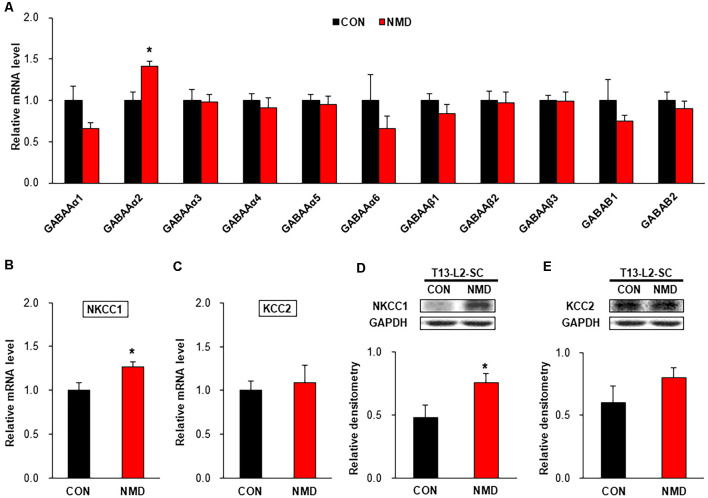
NMD enhanced sodium-potassium chloride cotransporter 1 (NKCC1) expression. **(A)** The mRNA level of GABA receptors in the spinal dorsal horn of T13-L2 in NMD rats compared with CON rats (*P < 0.05, *n* = 4 rats for each group, two-sample *t*-test). **(B)** The mRNA level of NKCC1 in the spinal dorsal horn of T13-L2 in NMD rats compared with CON rats (*P < 0.05, *n* = 4 rats for each group, two-sample *t*-test). **(C)** The mRNA level of KCC2 in the spinal dorsal horn of T13-L2 in NMD rats compared with CON rats (*P* > 0.05, *n* = 4 rats for each group, two-sample *t*-test). **(D)** Expression of NKCC1 in the spinal dorsal horn of T13-L2 in NMD rats compared with CON rats (*P < 0.05, *n* = 6 rats for each group, two-sample *t*-test). **(E)** Expression of KCC2 in the spinal dorsal horn of T13-L2 in NMD rats compared with CON rats (*P* > 0.05, *n* = 6 rats for each group, two-sample *t*-test).

To investigate the mechanism of decreased sIPSCs in NMD rats, we detected the expression of GABA receptors, NKCC1, and KCC2. As shown in Figure 5A, the mRNA level of GABA_A_α2 was significantly increased compared with CON rats, while other GABA receptors were not changed in T13-L2 spinal dorsal horn of NMD rats (Figure 5A, *P < 0.05, two-sample *t*-test). The mRNA level of NKCC1 was significantly increased compared with CON rats (Figure 5B, *P < 0.05, two-sample *t*-test), but the mRNA level of KCC2 was not altered in the T13-L2 spinal dorsal horn of NMD rats (Figure 5C, *P* > 0.05, two-sample *t*-test). Further, the relative expression levels of NKCC1 protein in the spinal dorsal horn of NMD rats were significantly increased compared with CON rats, suggesting a possible involvement of NKCC1 in IPSC and visceral pain (Figure 5D, *P < 0.05, two-sample *t*-test). However, the relative expression levels of KCC2 protein in the spinal dorsal horn of NMD rats was not altered compared with CON rats (Figure 5E, *P* > 0.05, two-sample *t*-test).

### Bumetanide Attenuated Chronic Visceral Pain of NMD Rats *via* a Postsynaptic Mechanism

**Figure 6:**
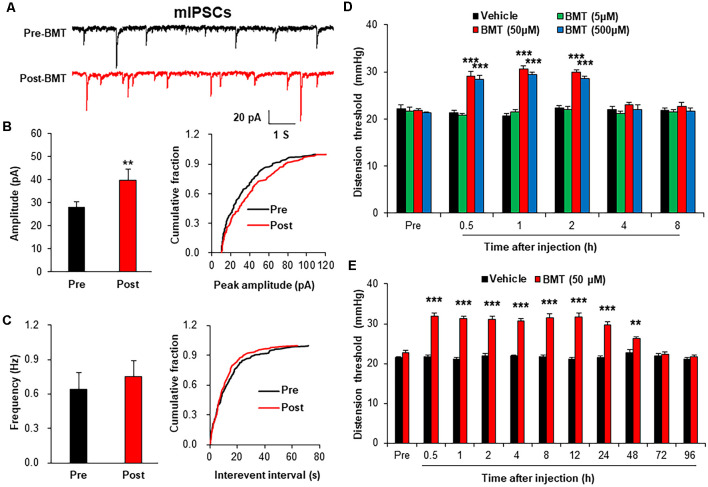
Bumetanide (BMT) enhanced spinal mIPSCs and visceral pain threshold in NMD rats. **(A)** Representative traces of mIPSCs were recorded in spinal dorsal horn neurons of NMD rats before and after incubation of BMT. **(B)** Bar plots and cumulative probability distributions of the amplitude of mIPSCs recorded in spinal dorsal horn neurons of NMD rats before and after incubation of BMT (**P < 0.01, *n* = 6 cells for each group, two-sample *t*-test). **(C)** Bar plots and cumulative probability distributions of the frequency of mIPSCs recorded in spinal dorsal horn neurons of NMD rats before and after incubation of BMT (*P* > 0.05, *n* = 6 cells for each group, two-sample *t*-test). **(D)** Behavioral changes of NMD rats after intrathecal injection of BMT at different doses (5, 50, and 500 μM, ***P < 0.001, *n* = 6 rats for each group, Tukey *post hoc* test following two-way repeated-measures ANOVA). **(E)** Behavioral changes of NMD rats after intrathecal injection of BMT (50 μM) for seven consecutive days (**P < 0.01, ***P < 0.001, *n* = 6 rats for each group, Tukey *post hoc* test following two-way repeated-measures ANOVA).

To evaluate the role of NKCC1 in inhibitory synaptic transmission, mIPSCs were recorded after incubation of BMT (20 μM), a selective inhibitor of NKCC1. The representative trace of a typical neuron showed that suppression of NKCC1 by BMT increased the amplitude of mIPSCs recorded in spinal dorsal horn neurons of NMD rats (Figure 6A). The Amplitude of mIPSCs in Post-BMT was significantly increased compared with Pre-BMT (Figure 6B, **P < 0.01, two-sample *t*-test), but the frequency of mIPSCs in Post- BMT was not altered (Figure 6C, *P* > 0.05, two-sample *t*-test). The result of mIPSCs suggests that NKCC1 decreased the inhibitory synaptic transmission of spinal dorsal horn neurons of NMD rats *via* a postsynaptic mechanism.

To further evaluate the role of NKCC1 in chronic visceral pain, we detected the CRD threshold of NMD rats after intrathecal injection of BMT. After a single injection of DMSO or BMT (5, 50 and 500 μM) in NMD rats, the threshold of colorectal distention in NMD+BMT (50 and 500 μM) was significantly increased compared with NMD + DMSO, but the threshold of colorectal distention in NMD+BMT (5 μM) was not altered (Figure 6D, ***P < 0.001, Tukey *post hoc* test following two-way repeated-measures ANOVA). Therefore, BMT (50 μM) was selected as the optimal concentration for the long-term behavioral test. After daily injection of DMSO or BMT (50 μM) in NMD rats for a week, the threshold of colorectal distention in NMD+BMT was significantly increased compared with NMD + DMSO (Figure 6E, **P < 0.01, ***P < 0.001, Tukey *post hoc* test following two-way repeated-measures ANOVA). These results suggest that NKCC1 contributes to chronic visceral pain of NMD rats.

### ASIC1 Upregulation of NKCC1 Expression Contributes to Visceral Pain in NMD Rats

**Figure 7:**
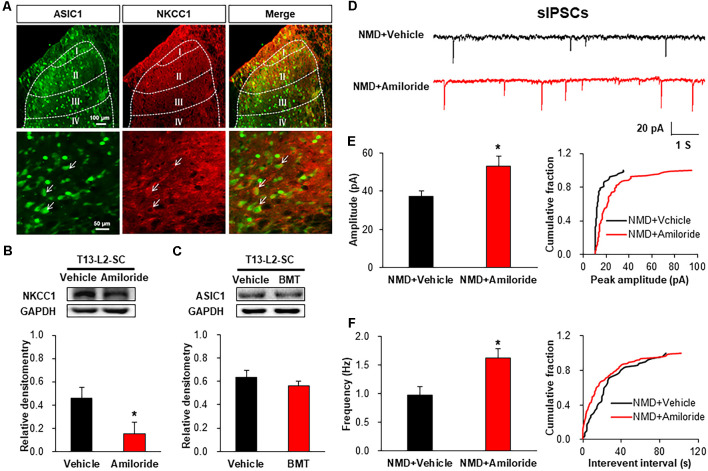
Interaction of ASIC1 and NKCC1. **(A)** ASIC1 and NKCC1 were co-located in the spinal dorsal horn neurons. **(B)** Expression of NKCC1 in the spinal dorsal horn after intrathecal injection of Amiloride in NMD rats (*P < 0.05, *n* = 6 rats for NMD + DMSO group and *n* = 5 rats for NMD+Amiloride group, two-sample *t*-test). **(C)** Expression of ASIC1 in the spinal dorsal horn after intrathecal injection of BMT in NMD rats (*P* > 0.05, *n* = 4 rats for each group, two-sample *t*-test). **(D)** Representative traces of sIPSCs recorded in spinal dorsal horn neurons of NMD rats after injection of Amiloride and DMSO. **(E)** Bar plots and cumulative probability distributions of the amplitude of sIPSCs recorded in spinal dorsal horn neurons of NMD rats after injection of Amiloride and DMSO (*P < 0.05, *n* = 6 cells for NMD + DMSO group and *n* = 7 cells for NMD+Amiloride group, two-sample *t*-test). **(F)** Bar plots and cumulative probability distributions of the frequency of sIPSCs recorded in spinal dorsal horn neurons of NMD rats after injection of Amiloride and DMSO (*P < 0.05, *n* = 6 cells for NMD + DMSO group and *n* = 7 cells for NMD+Amiloride group, two-sample *t*-test).

Immunofluorescence assay showed that ASIC1 was co-localized with NKCC1 in the spinal dorsal horn, suggesting a probable regulatory relationship between them (Figure 7A). Further, the protein level of NKCC1 was significantly decreased after Amiloride treatment (Figure 7B, *P < 0.05, two-sample *t*-test). However, the ASIC1 expression was not altered after injection of BMT (Figure 7C, *P* > 0.05, two-sample *t*-test). Importantly, the representative traces of sIPSCs in NMD rats showed that inhibitory synaptic transmission was significantly enhanced after Amiloride treatment (Figure 7D). The amplitude and frequency of sIPSCs in NMD+Amiloride were significantly increased compared with NMD + DMSO (Figures 7E,F, *P < 0.05, two-sample *t*-test). These results indicate that Amiloride enhanced sIPSCs might by downregulating NKCC1.

## Discussion

In the present study, we demonstrated that the simulation of the NMD induced chronic visceral pain in adult rats, which is consistent with the published articles from our lab and others (Chen et al., 2017; Du et al., 2019; Li et al., 2020). Importantly, we showed that both ASIC1 and NKCC1 in the spinal dorsal horn were involved in chronic visceral pain *via* increased excitatory synaptic transmission in the NMD rats. Activation of ASIC1 leads to Ca^+^ /Na^+^ influx, which leads to depolarization of neurons, thus enhancing excitability. NKCC1 expression was increased in the T13-L2 segments of the spinal cord in NMD rats, which maintained a high concentration of intracellular Cl^−^ in the neurons. Under this condition, activation of GABA receptors leads to the outflow of Cl^−^, which causes the depolarization of neurons, thus enhancing the excitability of neurons. Also, it should be noted that ASIC1 reduced inhibitory synaptic transmission by upregulation of NKCC1, thereby leading to the development of chronic visceral pain in NMD rats. Our findings suggest targeting the ASIC1-NKCC1 signaling pathway might be a potential strategy to treat visceral pain in patients with IBS.

Recent studies have shown that ASIC1 was involved in the regulatory mechanisms of inflammatory pain and migraine (Fu et al., 2016; Verkest et al., 2018; Li H.-S. et al., 2019). Our previous work has shown that ASIC1 was involved in gastric pain in adult rats with prenatal maternal stress (PMS; Wang et al., 2020). In the present study, we demonstrated that ASIC1 at the spinal cord level mediates chronic visceral pain in another rat model, the NMD rat model. The western blot results showed that only ASIC1 expression was significantly up-regulated in the spinal dorsal horn of NMD rats, and the visceral pain in NMD rats was significantly reversed by the injection of Amiloride. In the present study, we focused on the ASIC1 subtype but did not identify the expression of the specific subunit of ASIC1. However, based on available literature and our results, we speculate that ASIC1a regulates visceral pain in NMD rats, with the following evidence: (1) intrathecal injection of PcTX1, a specific inhibitor of ASIC1a, significantly increased the CRD threshold of NMD rats. (2) Previous studies have shown that ASIC1a rather than ASIC1b was primarily expressed at the spinal cord level (Chen et al., 2002; Wu et al., 2004; Wang et al., 2006; Lingueglia, 2007). However, we still could not completely rule out the role of ASIC2, because Amiloride was a non-specific inhibitor of ASICs. Also, we showed that the amplitude and frequency of sEPSCs in NMD rats were significantly enhanced, while the amplitude of mEPSCs was significantly reduced but the frequency was not altered by Amiloride, suggesting that ASIC1 plays a role through the post-synaptic mechanism. It should be noted that sIPSCs in the dorsal horn neurons were significantly reduced in NMD rats after the injection of Amiloride, which strongly suggests that ASIC1 may be involved in regulating sIPSCs in some way. Of note is that, we were unable to make sure those recorded neurons were required to transmit chronic visceral pain under the current recording conditions. Neurons recorded in the present study might include neurons that may not innervate the colon.

A potential mechanism by which ASIC1 regulates the sIPSCs is realized by NKCC1. As a Cl^−^ transporter, NKCC1 plays an important role in neuropathic pain (Modol et al., 2014; Yousuf and Kerr, 2016; Yousuf et al., 2017; Li C. et al., 2019) and bone cancer pain (Gao et al., 2019). Importantly, we provided new evidence to confirm that NKCC1 was involved in chronic visceral pain. This conclusion was based on the following evidence. NKCC1, rather than KCC2, was significantly up-regulated in the spinal dorsal horn of NND rats. Intrathecal injection of BMT, an inhibitor of NKCC1, significantly reversed visceral pain, indicating that NKCC1 was involved in chronic visceral pain in NMD rats.

To further investigate the interaction of ASIC1 and NKCC1, an immunofluorescence assay was carried out. Results showed that ASIC1 and NKCC1 were co-expressed in spinal dorsal horn neurons, suggesting that there is an anatomic base for the possible interaction between ASIC1 and NKCC1. Since incubation of BMT significantly reduced the amplitude rather than the frequency of mIPSCs decreased, it is reasonable to conclude that NKCC1 also functions through a postsynaptic mechanism. This is consistent with the mechanism of the ASIC1 role. The above data suggest that NKCC1 is involved in chronic visceral pain *via* reduces inhibitory synaptic transmission of spinal dorsal horn neurons in NMD rats. To determine the regulatory relationship between ASIC1 and NKCC1, intrathecal injection of inhibitors of ASIC1 or NKCC1 was used. The injection of Amiloride significantly reversed NKCC1 expression, indicating that ASIC1 might be an upstream regulator of NKCC1 expression. However, the expression of ASIC1 was not altered after the injection of BMT, further support the above idea. The detailed mechanisms by which ASIC1 regulates NKCC1 expression remain largely unknown. According to available references, there might be indirect and direct pathways involved in this regulation. Since ASIC1 regulates brain-derived neurotrophic factor (BDNF) expression (Coryell et al., 2009; Wang et al., 2018) and BDNF regulates NKCC1 expression and synaptic transmission (Cramer et al., 2008; Lu et al., 2009; Boulenguez et al., 2010; Hasbargen et al., 2010), we speculate that ASIC1 might indirectly regulate NKCC1 expression through BDNF. This needs to be confirmed in future studies. Although more experiments are needed, our data provide new evidence to demonstrate that ASIC1 regulates NKCC1 expression.

In summary, the present study demonstrated that ASIC1 regulates the expression of NKCC1 in the spinal dorsal horn, which is manifested as spinal sensitization on the whole, eventually leading to chronic visceral pain in NMD rats. These findings are expected to provide new therapeutic directions and ideas for the treatment of chronic visceral pain in patients with IBS.

## Data Availability Statement

The raw data supporting the conclusions of this article will be made available by the authors, without undue reservation.

## Ethics Statement

The animal study was reviewed and approved by Institutional Animal Care and Use Committee of Soochow University.

## Author Contributions

Y-CL performed the experiments, analyzed the data, and prepared figures and the manuscript. Y-QT, Y-YW, and Y-CX performed the experiments and analyzed the data. P-AZ and JS analyzed data and revised the manuscript. G-YX designed and supervised the experiments and finalized the manuscript. All authors contributed to the article and approved the submitted version.

## Conflict of Interest

The authors declare that the research was conducted in the absence of any commercial or financial relationships that could be construed as a potential conflict of interest.
